# Caught in the Act: Tumor-Immune Interactions in Circulation of Patients with Immune Marker Positive Circulating Tumor Cells

**DOI:** 10.3390/cancers17223667

**Published:** 2025-11-15

**Authors:** Amin Naghdloo, Mohamed Kamal, Dean Tessone, Valerie Hennes, James Hicks, Peter Kuhn

**Affiliations:** 1 Convergent Science Institute in Cancer, University of Southern California, Los Angeles, CA 90089, USA; naghdloo@usc.edu (A.N.); salehm@usc.edu (M.K.); tessone@usc.edu (D.T.); vhennes@usc.edu (V.H.); 2 Department of Aerospace and Mechanical Engineering, University of Southern California, Los Angeles, CA 90089, USA; 3 Department of Biological Sciences, University of Southern California, Los Angeles, CA 90089, USA; 4 USC Norris Comprehensive Cancer, Keck School of Medicine, University of Southern California, Los Angeles, CA 90089, USA; 5 Department of Biomedical Engineering, University of Southern California, Los Angeles, CA 90089, USA; 6 Department of Urology, Keck School of Medicine, University of Southern California, Los Angeles, CA 90089, USA

**Keywords:** tumor-immune interaction, liquid biopsy, circulating tumor cells, large extra-cellular vesicles, trogocytosis, CD4+ T cells

## Abstract

Background/Objectives: Circulating tumor cells (CTCs) and large extracellular vesicles (LEVs) are key components of the liquid biopsy that provide minimally invasive access to tumor biology. A clinically relevant subset of CTCs coexpressing epithelial and immune markers (im.CTCs) has been described, yet the origin of this phenotype remains unclear. In this study, we investigated the cellular and molecular context underlying the emergence of immune marker expression on CTCs and LEVs. Methods: Using high-resolution immunofluorescence microscopy of patient-derived blood samples, we identified direct physical interactions between white blood cells (WBCs) and both im.CTCs and im.LEVs, exclusively in patients harboring im.CTCs. Results: In several cases, WBCs partially encapsulated CTCs and LEVs, and quantitative analysis revealed localized enrichment of immune membrane markers at the contact interface, distinguishing these events from random proximity. Proteomic profiling further identified CD4+ T cells as the predominant interacting immune cell type and confirmed the presence of CD45, CD3, and CD4 on the interacting CTCs and LEVs, matching their WBC counterparts. Conclusion: These findings support membrane transfer as a potential mechanism for the acquisition of immune markers by CTCs and LEVs and provide in vivo evidence of contact-dependent tumor-immune interactions in circulation with implications for immune modulation and clinical interpretation of the im.CTC phenotype.

## Simple Summary

Liquid biopsy enables the study of cancer through blood samples by examining circulating tumor cells (CTCs) and large extracellular vesicles (LEVs). A subset of CTCs expressing immune markers has been reported, but the basis for this phenotype has remained unclear. In this study, we analyzed patient blood samples using high-resolution immunofluorescence imaging to investigate how tumor cells and vesicles may acquire these immune features. We observed direct physical interactions between white blood cells (WBCs) and the CTCs or LEVs that expressed immune markers, and such interactions occurred exclusively in patients who harbored these immune-marker-positive CTCs. In many instances, WBCs partially enveloped the tumor cells or vesicles, and immune markers were specifically concentrated at the regions of contact. Proteomic analysis identified CD4+ T cells as the predominant interacting immune cell type and confirmed that the same immune markers present on these WBCs were also found on the interacting CTCs and LEVs. These results support membrane transfer during cell-to-cell contact as a plausible mechanism for the acquisition of immune markers and provide in vivo evidence of contact-dependent tumor-immune interactions in circulation.

## 1. Introduction

Liquid biopsy provides a minimally invasive strategy for cancer profiling by analyzing tumor-derived material in body fluids, predominantly peripheral blood [1]. Among the key tumor analytes are circulating tumor cells (CTCs) and large extracellular vesicles (LEVs), which serve as biomarkers for cancer diagnosis, prognosis, and treatment monitoring [2,3,4]. CTCs detach themselves from primary or metastatic tumors and enter the bloodstream, where they can extravasate at a distant site and seed metastatic lesions. In epithelial malignancies, conventional CTCs are identified as cells that express epithelial markers, such as epithelial cell adhesion molecule (EpCAM) and/or cytokeratin (CK), and lack pan-leukocyte marker CD45 [5]. To this general CTC phenotype, we can apply genomic analysis using single-cell DNA sequencing to further characterize CTCs that share a common set of genetic markers typical of clonal tumor cells. In addition to epithelial CTCs, other CTC and tumor-associated phenotypes have been identified in the peripheral blood of cancer patients [6,7,8,9,10,11], including CTCs undergoing epithelial-to-mesenchymal transition [9], platelet-coated CTCs [6], circulating endothelial cells [10], cancer-associated macrophage-like cells [12], and cancer-associated fibroblasts [13].

Our group recently reported, in two late-stage metastatic breast cancer patients, on a novel phenotype of CTCs that, in addition to a strong CK signal, displayed a remarkable set of immune markers characteristic of CD4+ T-cells, including CD45, CD3, CD4, and the stemness marker CD44. Furthermore, these cells revealed clonal tumor-specific genomic copy number profiles identical to those of the conventional epithelial CTCs (epi.CTCs) in the same patients, simultaneously establishing their malignant origin and confirming the immune-like CTCs (im.CTCs) as a divergent clone in the tumor lineage [7]. Their immune-like proteomic profile and genomic clonality suggest that their emergence may be driven either by transcriptional reprogramming [14] involving coordinated activation of multiple genes or by exchange of molecular material between T cells and tumor cells [15,16].

One proposed mechanism for the emergence of these immune-like CTCs is the transfer of membrane fragments from immune cells to tumor cells. This has been demonstrated in vitro, where tumor cells obtain immune surface markers including CD45 [15,17,18]. However, direct evidence of interactions between CTCs and immune cells in the peripheral blood of cancer patients remains limited. To date, such interactions were often characterized with limited detail in the context of circulating tumor-immune clusters. Liquid biopsy studies on heterotypic clusters of CTCs and white blood cells (WBCs) have primarily focused on their prognostic value and cellular composition [19,20,21,22], with minimal focus on active interactions or membrane marker exchange at the contact interface.

Knowing that im.CTCs share clonal genomic alterations with epi.CTCs in a subset of studied patients, we sought to investigate whether direct contact with immune cells could explain the acquisition of immune-like phenotypes in the broader im.CTC population. In this study, we provide evidence of direct contact-dependent interaction between CTCs and WBCs in two metastatic breast cancer patients presenting clonally altered im.CTCs. Building on the previous study by our group [7], we further compared the phenotype distribution of LEVs to that of epi.CTCs and im.CTCs, and discovered direct interactions between LEVs and WBCs as well. These interactions were characterized by partial encapsulation of im.CTCs and im.LEVs by WBCs and proteomic signatures implicating CD4+ T cells as frequent partners. The phenotypic similarity of im.CTCs and im.LEVs to CD4+ T cells supports a model in which contact-dependent interactions, potentially involving trogocytosis, contribute to the acquisition of immune markers. Together, these findings establish patient-derived evidence for a contact-mediated mechanism modulating circulating tumor phenotypes and highlight their implications for liquid biopsy interpretation and immune-targeted therapies for a distinct patient population.

## 2. Materials and Methods

### 2.1. Patient Samples and Study Cohorts

This novel analysis was performed on stored image data obtained from de-identified samples obtained in the course of previously published translational studies with no new patient recruitment or sample collection. In total, 148 patients with metastatic breast cancer were included in the enumeration analysis. These samples were selected as subsets from 3 different studies (54 patients from IRB # UP-17-00882 approved on 28 March 2024 [23], 27 patients from study IRB # UP-14-00523 approved on 21 October 2014, HS-16-00435 approved on 1 February 2019 [24], and 67 patients from study IRB # UP-14-00592 approved on 1 December 2014 [25]). All patient samples used in this study were originally collected under IRB-approved protocols at the respective clinical recruitment sites as described in the cited publications. These studies were conducted in accordance with the Declaration of Helsinki and were approved by the Institutional Review Board at the University of Southern California. The IRB approvals listed for USC cover data usage and downstream experimental analyses only, and the present study involves secondary analysis of previously generated data.

### 2.2. Sample Collection and Processing

As previously published, all de-identified samples consisted of 8 mL peripheral blood shipped in 10 mL Streck Cell-Free DNA BCT tubes at room temperature (RT) and processed within 48 h of collection. Sample processing was performed as previously described [26]. Briefly, upon receipt, total cell counts were measured using a hematology analyzer to determine the appropriate volume required to plate approximately 3 million cells per slide. Red blood cells were lysed via isotonic ammonium chloride lysis (A649-3, Thermo Fisher Scientific, Waltham MA, USA), and the remaining nucleated cells were deposited onto cell-adhesive glass slides (Marienfeld, Lauda, Germany), yielding between 8 and 16 slides per sample depending on initial cellular content. Slides were coated with 7% BSA, air-dried, and stored at −80 °C for downstream analysis [24,27].

### 2.3. Immunofluorescence Staining

Slides were thawed at RT for one hour and stained using an autostainer (IntelliPATH FLX, Biocare Medical LLC, Pacheco, CA, USA) following a previously described four-color IF staining protocol [6]. Briefly, cells were fixed with 2% paraformaldehyde (Electron Microscopy Sciences, Hatfield, PA, USA) for 20 min. Slides were incubated with 10% filtered goat serum (MilliporeSigma, Burlington, MA, USA) for 20 min at RT to block nonspecific binding sites. Slides were then incubated for 4 h with 2.5 µg/mL AlexaFluor^®^ 647-conjugated anti-CD31 (BioRad Laboratories, Irvine, CA, USA) and 100 µg/mL goat anti-mouse IgG Fab fragments (Jackson ImmunoResearch, West Grove, PA, USA). Cells were subsequently permeabilized with 100% methanol (ThermoFisher Scientific, Waltham, MA, USA) for 5 min. Slides were next incubated for 2 h at RT with a primary antibody cocktail including pan-cytokeratin (Sigma-Aldrich, St. Louis, MO, USA), CK19 (DakoAgilent, Santa Clara, CA, USA), AlexaFluor^®^ 488-conjugated anti-vimentin (Cell Signaling Technology, Danvers, MA, USA), and AlexaFluor^®^ 647-conjugated anti-CD45 (AbD Serotec, San Jose, CA, USA) in 10% filtered goat serum. After washing in TBS twice, slides were incubated with AlexaFluor^®^ 555-conjugated goat anti-mouse IgG1 (Invitrogen, Waltham MA, USA) secondary antibody at a 1:1000 dilution in 10% goat serum containing DAPI (Thermo Fisher Scientific, Waltham, MA, USA) for nuclear staining for 1 h at RT. Slides were then washed twice in TBS, mounted with a glycerol-based medium, and coverslipped for imaging. These slides were used for patient enumeration analysis and proteomic profiling. To confirm that the fluorescent signal in the AlexaFluor^®^ 647 channel was specific to the CD45 marker, an additional slide from patients with im.CTCs was stained as described above, using 0.2% Triton X-100 for permeabilization instead of methanol and omitting the CD31 and Vimentin antibodies. These slides were further used for high-resolution imaging.

### 2.4. Image Acquisition

Fluorescently labeled slides were scanned using a custom-built automated fluorescence scanning microscope equipped with a 10× objective, achieving an effective total magnification of 100×. For each of the four IF channels, as well as the optional brightfield channel, the scanner captured 2304 individual image frames per slide, generating an effective representation of a whole slide image (WSI). All acquired images were saved as 16-bit TIFF files.

### 2.5. Immunofluorescence Image Analysis

To identify CK+ events, including CTCs and LEVs, WSI data from patient samples were analyzed using a two-step strategy tailored to the characteristics of each population. For CTC identification, cells were segmented using a pre-trained segmentation model [11]. Objects with low or absent nuclear DAPI signal—defined as having DAPI intensity more than three standard deviations below the mean of all nucleated cells—were excluded to remove acellular structures, artifacts, and other non-nucleated events. The remaining nucleated cells were then filtered based on a CK intensity threshold of 6 standard deviations over the mean, calculated across the CK intensity distribution of all segmented cells according to previously established protocols [7,28]. Cells exceeding this threshold were selected as CTC candidates, and all of them were manually reviewed by human analysts for verification. For LEV identification, the same CK intensity threshold used in the CTC analysis was applied to segment the CK channel. Segmented events were filtered by applying a minimum size cutoff of 2.5 µm in diameter to exclude small debris and staining artifacts. To isolate LEVs as non-nucleated structures, events with high DAPI intensity were excluded, using the same DAPI threshold applied in the CTC identification. This filtering step yielded a pool of CK+, DAPI-candidate LEVs. Brightfield images were also acquired during whole-slide scanning to evaluate membrane structures and confirm LEVs as events with a clearly visible membrane in the brightfield image. All LEV candidates were manually reviewed by human analysts to confirm membranous structure and marker expression and avoid enumerating fluorescent artifacts.

### 2.6. Confocal Microscopy

Selected contacting tumor-immune events were further visualized using high-resolution confocal microscopy. The events were located on the slides and imaged via a laser-scanning confocal microscope (Zeiss 880 inverted microscope, Zeiss Corp., Oberkochen, Germany) using a 100× oil-immersion objective. Z-stack optical sectioning was performed at 0.2 µm intervals to resolve the three-dimensional localization of signals relative to CTCs and LEVs. Encapsulation features were defined as partial or asymmetric enrichment of the CD45 signal at the membrane interface between the tumor-derived structure and the contacting WBCs. Image analysis and three-dimensional projection were performed using the ImageJ software package (version 1.54j).

### 2.7. Targeted Proteomic Profiling with Imaging Mass Cytometry

Proteomic profiling of both standalone and in-contact CTCs, LEVs, and WBCs as target events was conducted using imaging mass cytometry (IMC) as previously described [29] to characterize the interaction. Target events were identified and located as regions of interest (ROIs) by IF staining and image analysis. Two slides (one from each patient) were then stained with a multiplexed cocktail of metal-conjugated antibodies targeting immune and epithelial proteins as provided in Table S1. ROIs were ablated and isotope signals were quantified using a CyTOF Helios machineStandard Biotools, South San Francisco, CA, USA), providing multichannel images, one for each ROI, with each channel corresponding to one marker. The image data were segmented with the default CellPose model, cyto3, and manually corrected via the CellPose graphical user interface to accurately capture LEVs, which are inherently difficult to segment and to adjust the misalignments in masks. After segmentation, target events together with a random selection of WBCs were labeled by matching their coordinates to coordinates from IF images. Mean ion counts were calculated and log-transformed to stabilize the variance. Hierarchical clustering was performed to capture distinct phenotypes of WBCs and tumor events.

### 2.8. Statistical Analysis and Data Visualization

**Figure 1:**
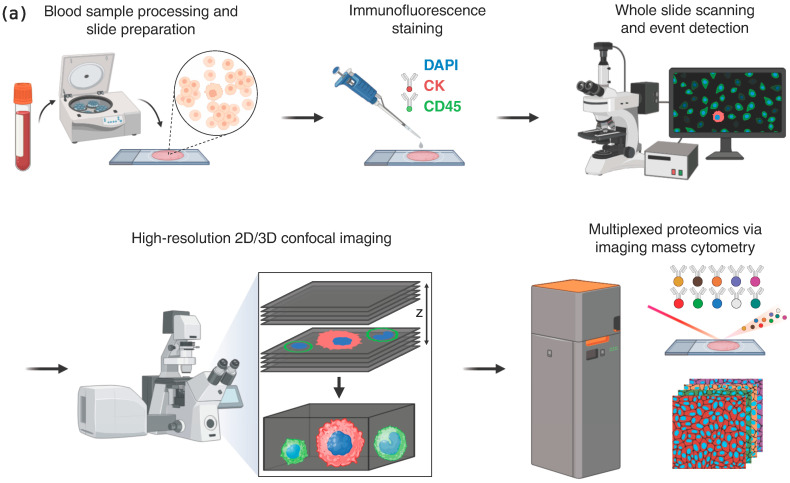

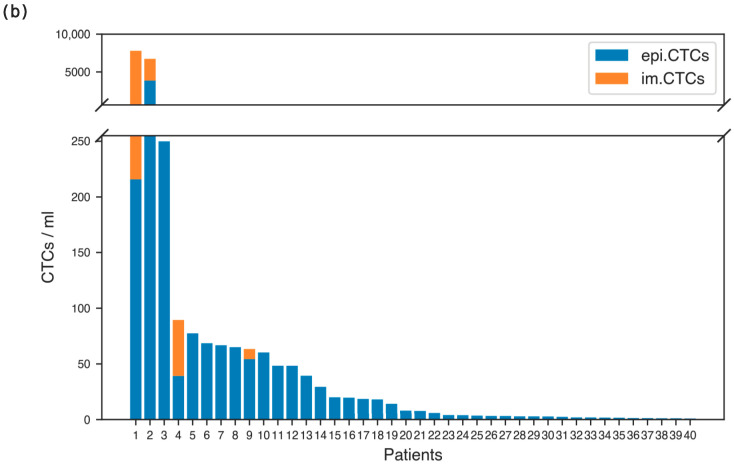
(**a**) Overview of analysis workflow. Cellular fractions were collected, plated on glass slides, and stained with an immunofluorescent assay including DAPI, CK, and CD45. Cell images were collected via whole slide scanning. Cells of interest were identified via image processing techniques and imaged at high resolution via widefield and confocal microscopy. Imaging mass cytometry was performed on events of interest to characterize their proteomic profile. (**b**) Enumeration of CTC phenotypes in metastatic breast cancer patients with CTCs present in their peripheral blood samples.

Statistical analysis, including Fisher’s exact test and Mann–Whitney U test, was performed via the scikit-learn package (v1.5.2). All data figures were created by visualization packages matplotlib (v3.5.1) and seaborn (0.13.2) in Python (3.9.0). The schematic in Figure 1 was created using the BioRender online software tool https://www.biorender.com (accessed on 12 June 2025).

### 2.9. Code Availability

The python package used for image analysis and visualization, slide-image-utils, is available at https://www.github.com/aminnaghdloo/slide-image-utils (accessed on 15 May 2025). The interactive tool used for enumeration of CTC and LEV phenotypes, annotateEZ, is available at https://www.github.com/aminnaghdloo/annotateEZ (accessed on 25 May 2025). All other analysis codes will be provided upon request.

## 3. Results

### 3.1. CTC and LEV Phenotype Enumeration Analysis in Metastatic Breast Cancer Patients

**Figure 2:**
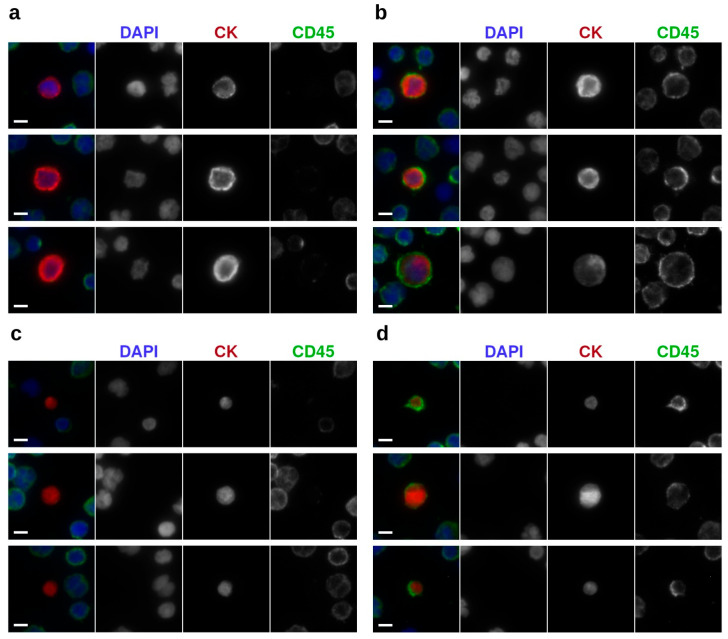
Representative images of tumor events with different phenotypes under 400× magnification: (**a**) epi.CTCs (**b**) im.CTCs; (**c**) epi.LEVs, and (**d**) im.LEVs. Scale bars: 5 µm.

To study the presence and frequency of the im.CTCs and epi.CTCs, peripheral blood samples from 148 patients with metastatic breast cancer were analyzed using our previously described enrichment-free liquid biopsy platform [6]. Figure 1a outlines the major steps of the workflow, from sample processing and staining to whole-slide imaging, automated detection, and high-resolution characterization, providing a visual summary of the analytical pipeline used in this study. Briefly, after red blood cell lysis, the remaining nucleated cells from each sample were plated on glass slides and stained with a multiplexed immunofluorescence panel designed to detect canonical and non-canonical CTC phenotypes based on the presence of CK, which all CTCs have in common. Detailed protocols for sample processing, staining, and image analysis are provided in the Methods. Both CTC phenotypes were identified based on their common expression of CK marker as described in Materials and Methods. Based on the enumeration results, 40 patients (27%) were found to have CTCs present in their blood. Figure 1b displays the distribution of total CTC counts and the relative proportions of epi.CTCs and im.CTCs per patient, illustrating the variability in CTC burden and the low prevalence of the im.CTC phenotype. We refer to the patients as P1–P40, ranked in descending order by the total number of CTCs present in each patient sample. Among these patients, only four (10%) exhibited the im.CTC phenotype (P1, P2, P4, and P9). All four patients were positive for hormone receptor and negative for human epidermal growth factor receptor 2 (HR+/HER2−). P1 and P2 had comparable but significantly higher CTC burden compared to P4 and P9, and each was explored in a different study [7,28]. The CTCs from patients P1 and P4 were previously studied [7], where they presented clonally altered genomic copy number profiles in both epi.CTC and im.CTC phenotypes, confirming that these im.CTCs are bona fide cancer cells. Figure 2a,b show representative examples of these two phenotypes, illustrating the characteristic CD45 expression patterns used to distinguish epi.CTCs and im.CTCs. In P1, the two draws collected three weeks apart showed no notable change in WBC count and the relative proportions of the two CTC phenotypes. However, in P2, 17 sequential draws over 4 years, captured periods of elevated CTC burden and fluctuating WBC counts in the context of a complex treatment regimen. These included one interval dominated by Estrogen receptor positive (ER+) CTCs with a very high fraction of im.CTCs that responded to therapy, and a later interval characterized by predominantly ER-CTCs with significantly lower im.CTC frequency, immediately preceding the patient’s passing. (Data in Table S3, and reference [28]).

In patients with im.CTCs as well as four additional patients for whom brightfield imaging data were available, we examined LEV phenotypes. Tumor-associated LEVs were identified and enumerated using the CK expression and brightfield imaging as described in Materials and Methods. Among these patients, the CD45 signal was detected in LEVs from four patients (P1–P4). Representative epi.LEVs and im.LEVs are shown in Figure 2c,d, respectively. These panels provide visual examples of the two LEV phenotypes, highlighting the presence or absence of the CD45 signal used for their classification. Notably, the fraction of im.LEVs were comparable to those of im.CTCs across analyzed patient samples containing both analytes (P1, P2, and P4). The complete distribution of LEV phenotypes across all patients with evaluable imaging data is summarized in Table 1.

### 3.2. Detection of Physiologically Interacting Tumor-Immune Clusters in Circulation

Given the emerging evidence that im.CTCs may arise through direct interactions with immune cells, we next sought to determine whether CTCs or LEVs in patient blood samples were physically associated with WBCs. From a total of 148 patients analyzed, we identified CTC-WBC neighbors, defined as any CTC and WBC whose segmented masks shared a boundary or overlapped in the whole-slide images. Because their abundance is affected by overall cell density on each slide, enumeration results were normalized to cell density to control for this effect. CTC-WBC neighbors were detected in 24 patients and were correlated with CTC abundance. Similarly, LEV-WBC neighbors were assessed in all patients with enumerated LEVs, and we found that they were all positive for LEV-WBC neighbors. The abundance of LEV-WBC neighbors showed a positive association with LEV abundance, in line with expected correlation and consistent with the trend observed for CTC-WBC neighbors (Figure S1).

**Figure 3:**
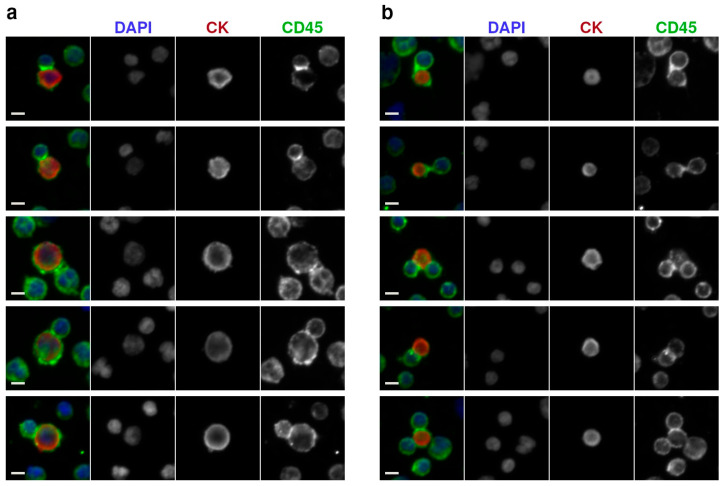
Detection of tumor-immune interacting events by high-resolution immunofluorescence microscopy (400× magnification): (**a**) Representative image of CTCs in physiological interaction with WBCs. (**b**) Representative image of large extracellular vesicles in contact with WBCs, showing epithelial marker positivity and localized CD45 signal at the interface and membrane. Scale bars: 5 µm.

High-resolution IF microscopy was then used to examine the tumor-immune neighbors for structural features consistent with active cellular engagement. Upon investigation, a subset of these neighbors was discovered with morphological characteristics indicative of potential biological interactions. This subset, referred to as physiologically interacting clusters (PICs), was annotated based on the morphology of the involved WBC resembling immune synapse polarization, with elevated CD45 expression at the contact interface, circumferential asymmetry oriented toward the CTC or LEV counterpart as well as their partial encapsulation by the WBC. Figure 3 shows representative examples of these events, demonstrating the structural criteria used to distinguish PICs.

Across the full cohort, PICs were observed in only two patients, P1 and P2 (Table 1). Both patients displayed im.CTCs and im.LEVs. PICs were absent in patients with either only im.CTCs (P9) or im.LEVs (P3) or none (P5–P8 and P10–P40). Additionally, both P1 and P2 had a high overall CTC burden (>6000 CTCs/mL). Importantly, PICs were absent in all other patients, including P4, with low CTC burden but presenting im.CTCs and im.LEVs as well as P3 with high CTC counts but lacking the im.CTC phenotype (Table 1).

### 3.3. PICs Are Not Attributable to Random Overlap

In samples generated by random plating of cell suspensions on glass slides, cells can overlap stochastically and may not reflect true biological interaction. We, therefore, set out to investigate whether the PICs observed in patients P1 and P2 reflect true biological interactions or random spatial coincidence. To address this, we examined a third patient, P3, who also exhibited a high number of tumor events (250 CTCs/mL and >1000 LEVs/mL). In this sample, 36 tumor-immune neighbors were identified that appeared to be overlapping at low magnification (100×). These events were then subjected to high-resolution imaging at 400× magnification to resolve physical boundaries and assess for features indicative of biological interaction.

**Figure 4:**
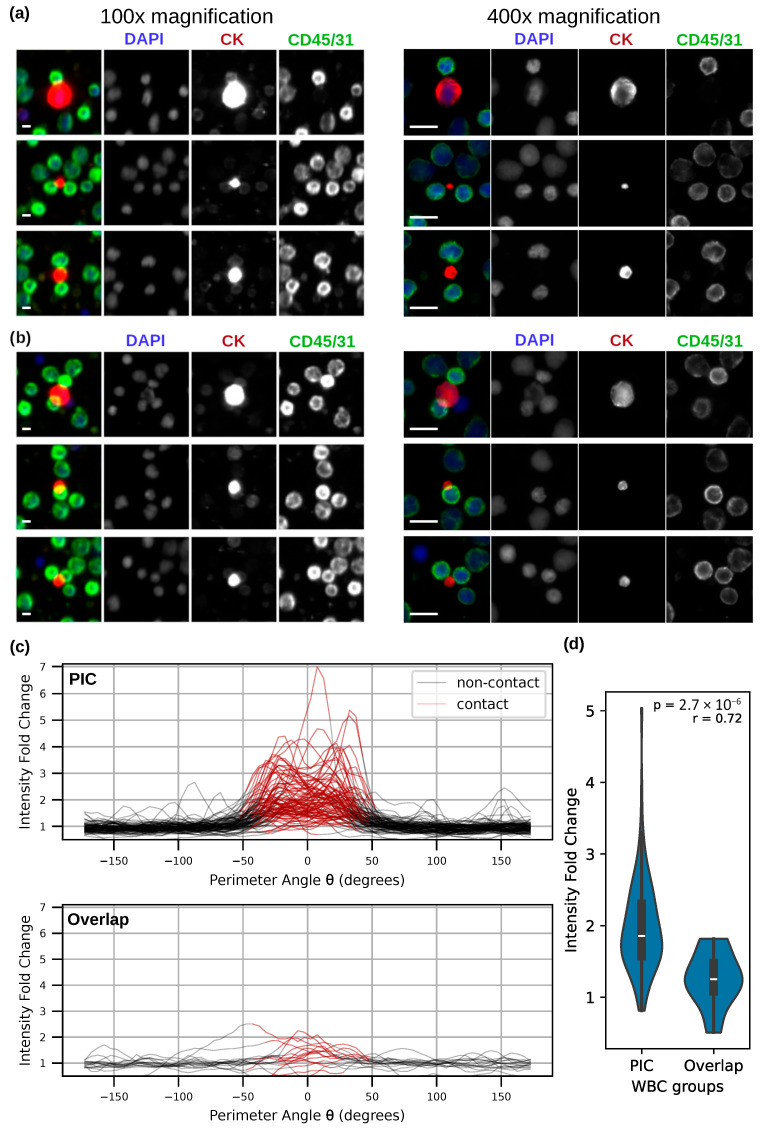
(**a**,**b**) Representative examples of overlapping tumor-immune neighbors in patient P3 under 100× (**Right**) and 400× (**left**) magnifications. Scale bars = 10 µm. (**a**) High-resolution imaging revealed the physical gap between 56% of the neighbors. (**b**) Overlapping WBCs in remaining neighbors retained intact morphology with no membrane deformation, distinguishing them from the PICs shown in Figure 3. (**c**) Membrane marker intensity changes along the WBC perimeter for PICs and overlapping neighbors. Each line represents an individual tumor-immune neighbor. (**d**) A Mann–Whitney U test of membrane intensity enrichment at contact regions for the two WBC groups.

Of the 36 identified neighbors, 20 (56%) were found to have visible physical gaps in between, indicating no direct contact. Figure 4a provides an example of this subset, where high-resolution imaging reveals a clear separation between the tumor event and the WBC despite apparent proximity at lower magnification. The remaining 16 neighbors appeared to be overlapping; however, they lacked key features seen in the PICs from P1 and P2. Notably, WBCs in overlapping neighbors retained an intact and rounded morphology and did not exhibit membrane signal enrichment or membrane deformation at the interface. Figure 4b shows overlapping neighbors that do not display morphological cues of cellular engagement, distinguishing them from the PICs shown in Figure 3.

To further quantify the membrane signal enrichment at the interface of CTC-WBC and LEV-WBC neighbors, we analyzed the intensity of the membrane marker along the perimeter of WBCs in high-resolution images from a subset of 93 PICs collected from P1 and all 16 overlapping neighbors from P3. Cell perimeters were defined from their segmented masks. Contact and non-contact regions for WBCs were defined as distinct sections of the cell perimeter, with contact regions defined as sections directly adjacent to the mask of the tumor event in the neighbors, and non-contact regions adjacent to the background. Intensity fold change was then calculated by comparing signal intensity between the contact and non-contact regions. Figure 4c depicts the intensity traces for individual contact pairs, illustrating the differences in contact-region enrichment between PICs and overlapping neighbors. PIC WBCs demonstrated statistically higher signal enrichment at the contact perimeter compared with overlapping WBCs (*p* = 2.76 × 10^−6^), with a large effect size (r = 0.71). Figure 4d summarizes this comparison using a Mann–Whitney U test, showing a clear separation between the two groups.

### 3.4. Confocal Imaging Reveals Spatial and Membrane Structural Features of PICs

**Figure 5:**
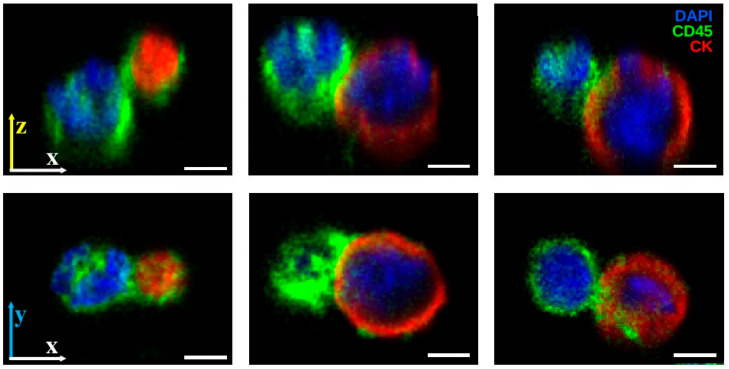
High-resolution confocal imaging of PICs. The CD45 (green) signal from WBC membranes extends partially over CK+ (red) CTCs or LEVs at the contact site, with enrichment at the interface in some cases. Three-dimensional reconstructions and x-z views show configurations where one cell is substrate-attached and the other is suspended above, supported by the contact interface. DAPI (blue) marks nuclei. Scale bars: 3 µm.

To further assess the observed PICs, high-resolution (1000× magnification) confocal microscopy was performed on a selected subset. Confocal images revealed WBC membrane extending partially over the target CTC or LEV at the contact site, with the CD45 marker localized on and projected over the CTC or LEV contact region. Figure 5 provides representative examples of these interactions, showing the partial membrane extension and localized CD45 signal at the interface that characterize PIC morphology. In several cases, this extension was asymmetric, with CD45 signal enriched at the contact interface. Three-dimensional reconstructions and lateral (x-z) views showed examples where only one cell involved in each PIC was in contact with the glass substrate, while the other was suspended above, supported by the contact interface. This spatial configuration supports the hypothesis that these cells are physically interacting, rather than merely positioned adjacent to one another. Supplementary Videos of 3D reconstructions and z-stacks provide an omnidirectional view of spatial orientation and tumor event interaction as well as spatial changes across the z coordinate.

### 3.5. Proteomic Analysis Reveals Prevalence of CD4+ T Cell Phenotype Among PICs

**Figure 6:**
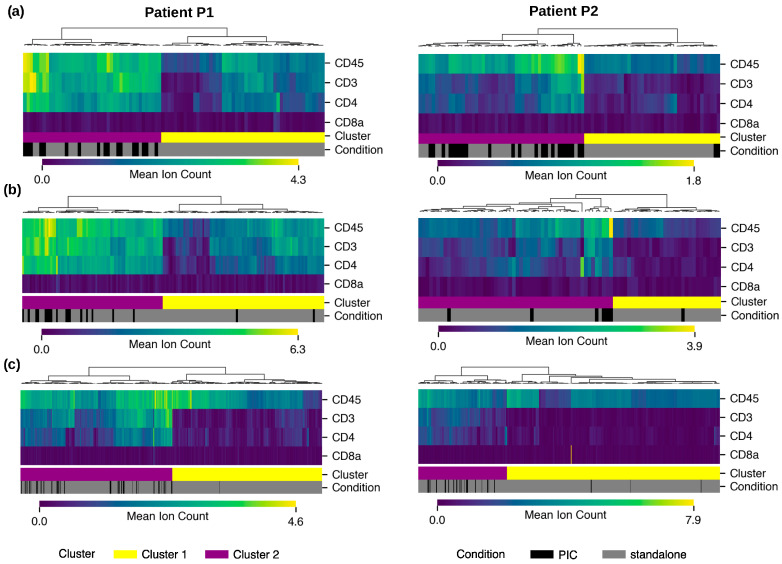
Proteomic profiling of PICs in two patients: Comparative analysis of marker prevalence in clusters of CD4+ T cell profile (cluster 1) versus the rest of WBCs (cluster 2) for (**a**) CTCs, (**b**) LEVs, and (**c**) WBCs. PICs are enriched in cluster 1 with a CD4+ T cell profile.

In order to further characterize PICs, proteomic analysis by imaging mass cytometry (IMC) was performed to explore immune markers. We previously explored the proteomic profile of standalone im.CTCs in patient P1 and reported high expression of multiple immune markers, including CD45, CD3, and CD4 in those cells compared to epi.CTCs [7], which is characteristic of CD4+ T cells. Across a total of 42 PIC CTCs and 143 standalone CTCs collected from patients P1 and P2, PIC CTCs were highly enriched with a proteomic profile resembling that of CD4+ T cells in both patients. Figure 6a illustrates this shift by showing the higher proportion of PIC CTCs mapping to the CD4+ T cell-associated cluster compared with standalone CTCs. Similarly, across 26 PIC LEVs and 219 standalone LEVs, PIC LEVs were highly enriched for the proteomic profile of CD4+ T cells. Figure 6b presents the corresponding distribution for LEVs, highlighting the preferential clustering of PIC LEVs within the CD4+ T cell-associated group.

To place these findings in the context of immune cell populations, we extended the proteomic analysis to PIC WBCs. Immune subtypes were determined via cell gating using CD45, CD3, CD4, CD8a, CD20, CD56, CD14, and CD68 (see details in Methods Section 2.7). Across the immune compartment, expression patterns indicated that PIC WBCs closely aligned with CD4+ T cells, displaying high levels of CD45, CD3, and CD4 expressions and low CD8 expression. Figure 6c shows this alignment by positioning PIC WBCs within the CD4+ T cell-associated cluster relative to another immune subset. Further, Fisher’s exact test was performed to quantify the significant prevalence of PICs in the CD4+ T cell phenotype group. Tumor events and immune cells were separately clustered based on CD4+ T cell markers into two main clusters, one representing the CD4+ T cell phenotype and the other representing any other event that lacks this phenotype. The statistical test showed that in contact events were significantly enriched in CD4+ T cell phenotype in cluster 1 compared to random distribution (Table S2).

### 3.6. Functional Protein Profiling in PICs

**Figure 7:**
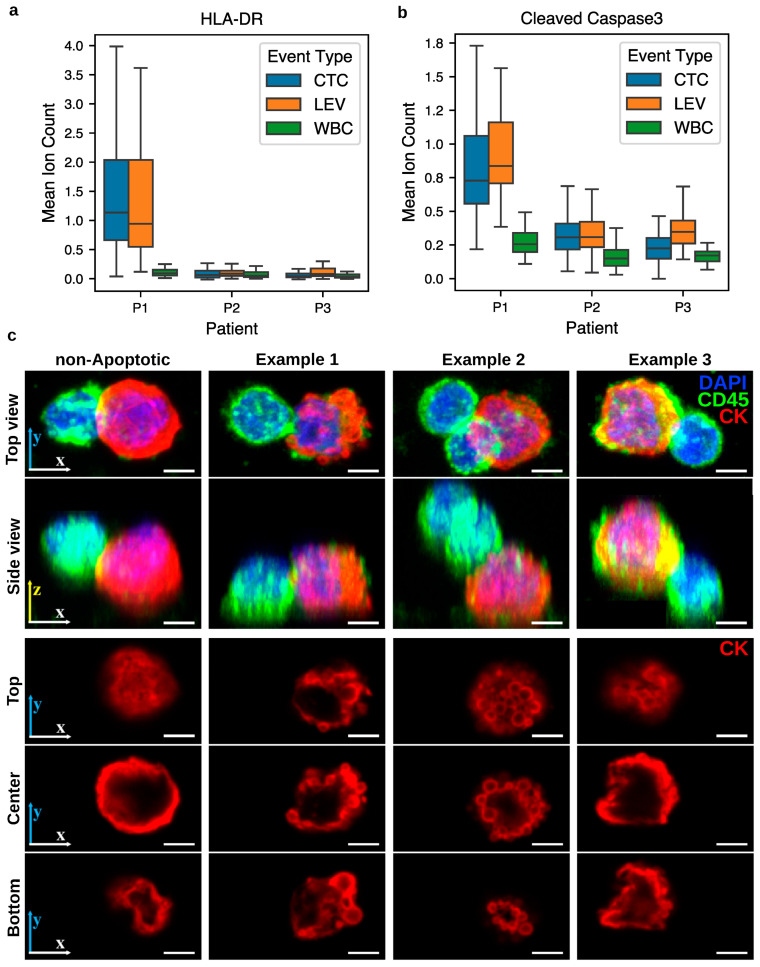
Functional protein expression patterns in CTCs and LEVs of patients P1 and P2 with PICs. (**a**) HLA-DR expression was elevated in CTCs and LEVs from patient P1 relative to WBCs, but not in patient P2. (**b**) Cleaved Caspase-3 was elevated in CTCs and LEVs from both patients, with higher levels in patient P1. (**c**) Confocal imaging of PIC CTCs shows three examples of blebbing morphology in the CTC (columns 2–4), compared to the PIC without such morphology (left). Rows 1–2 show 3D projections at two viewing angles, while rows 3–5 display cross-sections of the CK channel at 10%, 50%, and 90% of the total cell height. Scale bar: 3 µm.

Having established that PICs are enriched for a CD4+ T cell-like proteomic phenotype, we next examined selected functional protein markers to describe features present in these interactions beyond lineage characterization. Human Leukocyte Antigen-DR (HLA-DR), a Major Histocompatibility Complex Class II (MHC-II) molecule primarily targeted by CD4+ T cells, was elevated in both CTCs and LEVs of patient P1 compared to WBCs (Figure 7a), whereas no comparable elevation was observed in patient P2. In addition, cleaved Caspase-3, an apoptotic marker, was elevated in both CTCs and LEVs of patients P1 and P2, with a markedly stronger signal in P1 (Figure 7b). Confocal imaging of PIC CTCs revealed CTCs with blebbing morphology. Figure 7c demonstrates three such examples alongside a PIC without blebbing for comparison. While these observations are consistent with known features of immune-tumor cell interactions, they do not establish a mechanism. Rather, they highlight protein expression patterns present in PICs, which may serve as a basis for future functional studies.

## 4. Discussion

To our knowledge, this is the first study to provide in vivo evidence of direct physio-logical interaction and membrane exchange between tumor-derived structures and immune cells in the circulation of metastatic breast cancer patients presenting im.CTCs. Unlike previous studies that focused primarily on the enumeration and prognostic implications of circulating tumor-immune clusters [30,31], our work specifically analyzes the nature of the interaction at the single-cell level. Using high-resolution widefield and confocal immunofluorescence microscopy, we identified physiologically interacting clusters (PICs) of CTCs and LEVs that exhibit features consistent with active immune engagement, including immunological synapse-like membrane extensions from WBCs and localized enrichment of CD45 at the contact interface. The joint analysis of both im.CTCs and im.LEVs in this contact-dependent context distinguish our findings and suggest a mechanistic role for immune cells in shaping tumor phenotypes in circulation.

The presence of CD45+ CTCs has been increasingly recognized across multiple malignancies [32], but the biological mechanisms underlying the origin of this phenotype remain under investigation. Prior hypotheses have suggested cell fusion between tumor cells and macrophages [33] or uptake of immune cell-derived extracellular vesicles [16]. We previously reported identical genomic copy number alteration profiles between im.CTCs and epi.CTCs, detected in patients P1 and P4. This suggests that cell fusion is unlikely to be the origin of the observed im.CTCs in this patient population [7].

Findings from our deep characterization of physiological interactions suggest that im.CTCs and im.LEVs are produced as a product of trogocytosis. Trogocytosis is an active, contact-dependent process that allows for rapid acquisition of functional membrane proteins, relying on a dynamic interface between immune effectors and tumor cells that may contribute to phenotypic plasticity and immune evasion [34]. First, our comparative analysis of PICs and overlapping tumor-immune neighbors via high-resolution microscopy suggests that PICs are biologically interacting rather than random spatial overlap. Direct cell–cell contact is a hallmark of trogocytosis. Second, PIC CTCs were observed exclusively in patients who exhibited both im.CTCs and a very high overall CTC burden. This restricted distribution suggests that PICs are not simply a function of CTC abundance but are specifically associated with the concurrent presence of im.CTCs and elevated CTC load in circulation. This observation further indicates that these events are rare (3–4% of total CTCs) and may represent transient states with limited lifetime. This agrees with in vitro models of trogocytosis, which showed that tumor-immune contacts are short-lived and a rapid transfer of membrane signals can be detected within 10 min of cancer cell-T cell contact [15]. Third, proteomic analysis demonstrated enrichment of canonical CD4+ T cell markers (CD45, CD4, and CD3) among PICs, including LEVs, CTCs, and WBCs, compared to standalone events, suggesting that CD4+ T cells are involved in the observed interactions, potentially as donor cells in trogocytosis. CD4+ T cells have also been shown to engage in trogocytosis, rapidly transferring membrane proteins to interacting cells during short-lived contacts [15,17]. Moreover, recent work in clinical renal cell carcinoma samples has shown stable expression of CD45, CD14, CD16, and CD56 on tumor cells in situ, strongly implicating trogocytosis as an active mechanism of membrane acquisition during tumor-immune cell contact in patients [35].

Taken together, the morphological evidence of direct physiological interaction between cancer-immune cells neighbors, the exclusive presence of PICs in patients with high CTC burden and im.CTCs (which implies a transient contact), and the enrichment of CD4+ T cell markers within PICs [36,37], support trogocytosis as a plausible contact-dependent mechanism for the acquisition of immune membrane markers by CTCs. While trogocytosis has been documented in the tumor microenvironment [15], this study extends the evidence to patient blood samples. Whether trogocytosis occurs directly in the circulation or instead takes place within the tumor microenvironment prior to dissemination remains unresolved, and will require further functional studies.

In our study, PICs displayed CD4+ T cell-associated protein features and accompanying molecular and morphological patterns, such as HLA-DR expression in patient P1 and elevated cleaved Caspase-3 in both patients, that align with phenomena reported in immune-tumor interactions. Several models in the literature could be compatible with these observations, including MHC-II-mediated antigen recognition, CD4+ T cell cytotoxic activity, and other forms of immune signaling [38,39,40]. Additional mechanisms, such as trogocytosis or immune regulatory protein transfer under interaction conditions, have also been documented, implicating an immune evasion mechanism [15,17,41]. While our dataset does not allow us to distinguish among these possibilities, the fact that our observations are in line with several established models highlights the potential of PICs as a useful framework for studying rare immune-tumor interactions using patient blood samples. Future functional studies will be essential to differentiate between these models and determine which align most closely with our observations.

This study has a number of limitations. The observations are based on a small number of patient samples, reflecting the rarity of im.CTCs and tumor-immune contact events. Further, analyzing one slide per patient may limit the sensitivity in the detection of low-frequency events and potential temporal variation in samples. Enumeration of more than 1 slide (2.5 million cells on average) from each patient will increase the limit of detection for CTCs of both phenotypes, leading to more robust and unbiased patient statistics. As the analysis was limited to single-timepoint samples, temporal variability and clinical correlates of these events remain to be determined in larger studies with follow-up samples. While imaging and proteomic evidence support a model consistent with trogocytosis, functional assays, including CTC culture and deeper proteomic profiling of the interacting immune cells, will be required to definitively demonstrate membrane transfer and its mechanistic underpinnings. In future studies with larger cohorts, a panel of markers for functional characterization of PICs will be necessary to validate these findings and further elucidate the impact of tumor-immune interactions on cancer progression and immune modulation.

Together, our findings reveal a previously underappreciated dynamic between circulating tumor-derived structures and immune cells, expanding the landscape of tumor-immune interactions beyond the primary tumor microenvironment. The direct acquisition of immune markers by CTCs and LEVs via physical contact highlights a novel dimension of tumor cell plasticity in circulation and warrants further investigation into its biological significance and potential clinical implications. Beyond establishing the presence of these interactions, this study also demonstrates a framework for their functional characterization through high-resolution imaging and single-cell proteomics, thereby enabling direct investigation of the biological states and consequences of tumor-immune interactions in patients through the lens of liquid biopsy.

## 5. Conclusions

This study provides the first in vivo evidence that circulating tumor cells and large extracellular vesicles can directly engage with immune cells through contact-dependent mechanisms consistent with trogocytosis. By combining high-resolution imaging and single-cell proteomics, we uncovered physiologically interacting clusters that exhibit immune synapse-like structures and selective enrichment of CD4+ T cell markers, supporting a model in which immune membrane components are transferred to tumor-derived entities during transient interactions in circulation. These findings suggest that such interactions may influence both tumor plasticity and immune cell function. Ultimately, elucidating the mechanisms and consequences of these contact-dependent exchanges could offer new insights into metastatic progression and open avenues for developing therapeutic or diagnostic strategies that target the dynamic interface between cancer and the immune system in the bloodstream.

## Figures and Tables

**Table 1:** Enumeration results for standalone CTC and LEV phenotypes and their occurrence in physiologically interacting clusters (PICs) in patients with LEV data in count/mL.

Patient	im.CTCs	epi.CTCs	PIC CTCs	im.LEVs	epi.LEVs	PIC LEVs
P1	7582	216	278	9991	2521	501
P2	2872	3863	271	1097	4317	136
P3	0	250	0	2	2330	0
P4	50	39	0	61	128	0
P5	0	75	0	0	232	0
P8	0	65	0	0	725	0
P9	9	54	0	0	82	0
P12	0	48	0	0	58	0

## Data Availability

All data discussed in this manuscript are included in the main manuscript text or Supplementary Materials. The immunofluorescence images of circulating tumor cells (CTCs) and large extracellular vesicles (LEVs), along with their associated phenotypes as well as imaging mass cytometry (IMC) data, will be made publicly available through the BloodPAC Data Commons under the accession ID BPDC000155.

## Supplementary Materials

The following supporting information can be downloaded at: https://www.mdpi.com/article/10.3390/cancers17223667/s1, Figure S1. Frequency of CTC-WBC (left) and LEV-WBC neighbors (right) normalized by the cell density on the slide against overall abundance of the corresponding event type. Table S1: Antibody clones used in IMC experiment for proteomic profiling. Table S2: Statistical p-value results of enrichment analysis. Table S3: Longitudinal CTC, imCTC, and WBC measurements for Patients P1 and P2.
