# Supplementary material for: Caught in the Act: Tumor-Immune Interactions in Circulation of Patients with Immune Marker Positive Circulating Tumor Cells
Source: Cancers (Basel). 2025 Nov 15;17(22):3667. doi: 10.3390/cancers17223667 (PMC12651602; doi:10.3390/cancers17223667)
Supplement: Supplementary file 1 [file cancers-17-03667-s001.zip › supplementary_material/supplementary_file.pdf]

Supplementary Materials

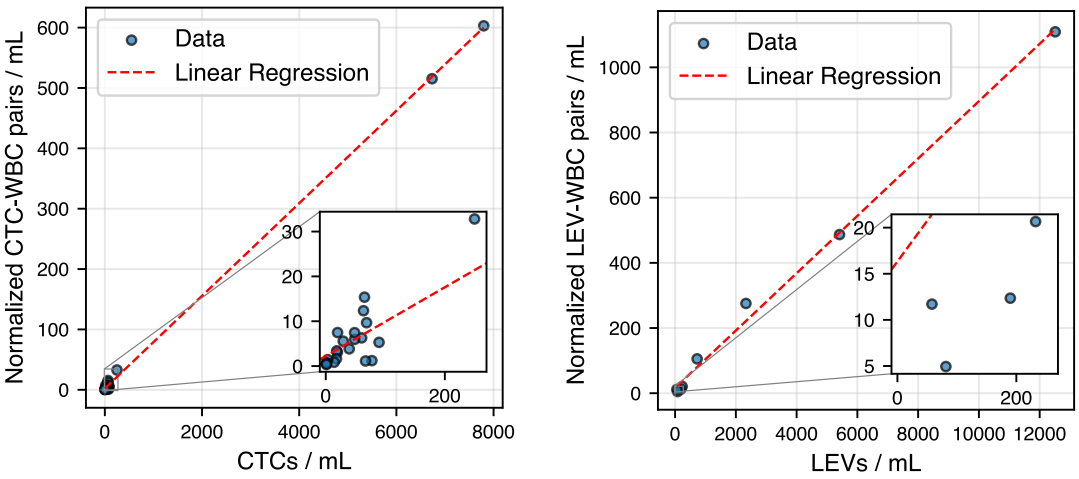

Figure S1. Frequency of CTC-WBC (left) and LEV-WBC neighbors (right) normalized by the cell density on the slide against overall abundance of the corresponding event type.

Table S1: Antibody clones used in IMC experiment for proteomic profiling

Target	Antibody Clone	Cell type/state
CK8	Rabbit_IgG_EP1628Y	Epithelial
CK18	Rabbit_IgG_EPR1626	Epithelial
CD45	Mouse_IgG1k_HI30	Immune cells
cleaved-caspase3	Rabbit_IgG_D3E9	Apoptotic cells
CD3	Rabbit_IgG_Polyclonal	T-cells
CD4	Rabbit_IgG_EPR6855	Helper CD4+ T-cells
CD8a	Mouse_IgG1k_RPA-T8	Cytotoxic CD8+ T-cells
HLA-DR	Mouse_IgG2ak_L243	Antigen presenting cells

Table S2: Statistical p-value results of enrichment analysis.

Patient	CTC	LEV	WBC
P1	2.0×10^{-7}	5.3×10^{-5}	3.6×10^{-10}
P2	1.0×10^{-5}	0.4	2.1×10^{-9}

Table S3: Longitudinal CTC, imCTC, and WBC measurements for Patients P1 and P2

Patient	Draw	Months from Baseline	imCTC/ml	Total CTC/ml	imCTC Fraction	WBC [$\times 10^6$/ml]
P1	1	0	7581	7797	0.97	5.9
P1	2	$<$1 (3 weeks)	6628	6731	0.98	5.4
P2	1	0	39	460	0.09	6.5
P2	2	2	64	207	0.31	7.6
P2	3	3	308	861	0.36	10.2
P2	4	7	2871	6734	0.43	11.0
P2	5	9	2946	52306	0.06	10.1
P2	6	12	382	1469	0.26	7.5
P2	7	14	0	1	0.00	2.2
P2	8	17	1	5	0.33	3.8
P2	9	20	0	0	0.00	4.4
P2	10	22	3	9	0.33	3.8
P2	11	24	4	6	0.67	3.5
P2	12	26	0	2	0.00	4.9
P2	13	31	59	194	0.31	3.3
P2	14	33	376	694	0.54	3.7
P2	15	35	23	155	0.15	6.2
P2	16	39	154	6640	0.02	5.8
P2	17	46	815	6823	0.12	10.8
